# Timeliness and Modality of Treatment for New Cancer Diagnoses During the COVID-19 Pandemic in Canada

**DOI:** 10.1001/jamanetworkopen.2022.50394

**Published:** 2023-01-10

**Authors:** Rui Fu, Rinku Sutradhar, Qing Li, Timothy P. Hanna, Kelvin K. W. Chan, Jonathan C. Irish, Natalie Coburn, Julie Hallet, Anna Dare, Simron Singh, Ambica Parmar, Craig C. Earle, Lauren Lapointe-Shaw, Monika K. Krzyzanowska, Antonio Finelli, Alexander V. Louie, Nicole J. Look Hong, Ian J. Witterick, Alyson Mahar, David R. Urbach, Daniel I. McIsaac, Danny Enepekides, Jill Tinmouth, Antoine Eskander

**Affiliations:** 1 ICES, Toronto, Ontario, Canada; 2 Institute of Health Policy, Management, and Evaluation, University of Toronto, Toronto, Ontario, Canada; 3 Department of Otolaryngology–Head and Neck Surgery, University of Toronto, Toronto, Ontario, Canada; 4 Division of Cancer Care and Epidemiology, Cancer Research Institute, Queen’s University, Kingston, Ontario, Canada; 5 Ontario Institute for Cancer Research, Toronto, Ontario, Canada; 6 Odette Cancer Centre–Sunnybrook Health Sciences Centre, Toronto, Ontario, Canada; 7 Ontario Health–Cancer Care Ontario, Toronto, Ontario, Canada; 8 Department of Otolaryngology–Head & Neck Surgery/Surgical Oncology, University of Toronto, Princess Margaret Cancer Centre, Toronto, Ontario, Canada; 9 Department of Surgery, University of Toronto, Toronto, Ontario, Canada; 10 Department of Medicine, University of Toronto, Toronto, Ontario, Canada; 11 Department of Radiation Oncology, University of Toronto, Toronto, Ontario, Canada; 12 School of Nursing, Queen’s University, Kingston, Ontario, Canada; 13 Department of Surgery, Women’s College Hospital, Toronto, Ontario, Canada; 14 Department of Anesthesiology and Pain Medicine, The Ottawa Hospital, Ottawa, Ontario, Canada

## Abstract

**Importance:**

The impact of COVID-19 on the modality and timeliness of first-line cancer treatment is unclear yet critical to the planning of subsequent care.

**Objective:**

To explore the association of the COVID-19 pandemic with modalities of and wait times for first cancer treatment.

**Design, Setting, and Participants:**

This retrospective population-based cohort study using administrative data was conducted in Ontario, Canada, among adults newly diagnosed with cancer between January 3, 2016, and November 7, 2020. Participants were followed up from date of diagnosis for 1 year, until death, or until June 26, 2021, whichever occurred first, to ensure a minimum of 6-month follow-up time.

**Exposures:**

Receiving a cancer diagnosis in the pandemic vs prepandemic period, using March 15, 2020, the date when elective hospital procedures were halted.

**Main Outcomes and Measures:**

The main outcome was a time-to-event variable describing number of days from date of diagnosis to date of receiving first cancer treatment (surgery, chemotherapy, or radiation) or to being censored. For each treatment modality, a multivariable competing-risk regression model was used to assess the association between time to treatment and COVID-19 period. A secondary continuous outcome was defined for patients who were treated 6 months after diagnosis as the waiting time from date of diagnosis to date of treatment.

**Results:**

Among 313 499 patients, the mean (SD) age was 66.4 (14.1) years and 153 679 (49.0%) were male patients. Those who were diagnosed during the pandemic were less likely to receive surgery first (subdistribution hazard ratio [sHR], 0.97; 95% CI, 0.95-0.99) but were more likely to receive chemotherapy (sHR, 1.26; 95% CI, 1.23-1.30) or radiotherapy (sHR, 1.16; 95% CI, 1.13-1.20) first. Among patients who received treatment within 6 months from diagnosis (228 755 [73.0%]), their mean (SD) waiting time decreased from 35.1 (37.2) days to 29.5 (33.6) days for surgery, from 43.7 (34.1) days to 38.4 (30.6) days for chemotherapy, and from 55.8 (41.8) days to 49.0 (40.1) days for radiotherapy.

**Conclusions and Relevance:**

In this cohort study, the pandemic was significantly associated with greater use of nonsurgical therapy as initial cancer treatment. Wait times were shorter in the pandemic period for those treated within 6 months of diagnosis. Future work needs to examine how these changes may have affected patient outcomes to inform future pandemic guideline development.

## Key Points

**Question:**

Is the COVID-19 pandemic associated with the modality of and wait times for treatment among patients with new cancer diagnoses?

**Findings:**

In this cohort study comprising 313 499 Canadian adults newly diagnosed with cancer in 2016 to 2020, those diagnosed during the pandemic were less likely to receive surgery as an initial treatment and were more likely to receive chemotherapy or radiation first during the first postdiagnosis year. Mean wait times decreased in the pandemic for each modality among those who were treated within 6 months.

**Meaning:**

The pandemic-associated shifts in cancer management observed in this study may reflect changes in the patient case-mix, which requires further understanding to minimize potential negative repercussions.

## Teaser

This cohort study explores the association of the COVID-19 pandemic with modalities of and wait times for first cancer treatment in Ontario, Canada.

## Introduction

There are growing concerns regarding disrupted cancer care during the COVID-19 pandemic that may lead to negative long-term patient outcomes.^1^ These concerns are accentuated for patients who were newly diagnosed with cancer in the midst of the pandemic as they tried to navigate an overwhelmed system to access their first cancer treatment. Shortly after the start of COVID-19, the weekly volume of cancer treatment, including surgery, chemotherapy, and radiation therapy, declined given that hospitals urgently diverted resources to create capacity for COVID-19 care.^2,3,4,5^ What has accompanied this decrease in supply of cancer treatment was a similarly lowered demand for these services, as the pause in cancer screening, imaging, and in-person oncologist visits^6,7^ led to fewer cancer diagnoses and a potentially increased presentation of inoperable advanced disease.^8,9^ At the system level, there has been a directed prioritization of cancer care, especially cancer surgical care, over other surgical and nonsurgical interventions for nononcologic patients, which could have created an expediated care pathway for patients with cancer.^10,11^ These complex shifts in cancer system capacity and patient profiles suggest that the pandemic may have had unique consequences for treatment modalities and wait times among patients with new cancer diagnoses, which requires a large population-based individual-level analysis to elucidate.^12,13^ Such an analysis, to our knowledge, is currently lacking in the literature. The objective of this study was to examine the association between the pandemic and the patterns of first cancer treatment, including modalities and wait times, in a cohort of adult patients newly diagnosed with cancer from 2016 to 2020.

## Methods

### Study Design

This population-based retrospective cohort study was based in Ontario, Canada, where universally accessible physician services are offered to 14.6 million residents through the Ontario Health Insurance Plan (OHIP).^14^ This study was approved by the Privacy and Legal Office of ICES. The use of data in this study is authorized under section 45 of Ontario’s Personal Health Information Protection Act and does not require review by a research ethics board. This study followed the Strengthening the Reporting of Observational Studies in Epidemiology (STROBE) reporting guideline.

### Data Sources

Administrative data sets (eTable 1 in Supplement 1) were linked using unique encoded identifiers and analyzed at ICES. The Ontario Cancer Registry (OCR) captures 96% of index cancers across the province.^15,16^ The Registered Persons Database (RPDB) maintains sociodemographic data of permanent residents. The Immigration, Refugees and Citizenship Canada (IRCC) Permanent Resident Database (with data from January 1985 to May 2017) includes records of individuals who immigrated to Ontario during this period. The OHIP claims database contains physician billing records. Canadian Institute for Health Information (CIHI) Hospital Discharge Abstract database and Same-Day Surgery database contain information on at-hospital surgery. Canadian census conducted by Statistics Canada was available to construct neighborhood rurality and material deprivation (extracted from the Ontario Marginalization Index), a validated measure of socioeconomic status.^17^

### Study Cohort

We used the OCR to identify adults who were diagnosed with cancer between January 3, 2016, and November 7, 2020 (eTable 2 in Supplement 1). The accrual end date reflects the last reliable update of cancer incidence data from the OCR at time of this analysis (January 2022). If more than 1 cancer diagnosis occurred in this period, only the date of the first diagnosis was selected. We excluded patients with a diagnosis of melanoma, skin cancer, ophthalmologic cancer, or paraneoplastic neurological syndromes (PNSs). Melanoma and skin cancer are more frequently treated in the outpatient setting, and as a result we could not ensure complete capture using information on hospital-based cancer surgeries. Exclusion of cancers primarily labeled as ophthalmologic and PNS were due to their rarity (<0.04%) (eFigure in Supplement 1). Patients were followed up from the date of cancer diagnosis (the date of specimen taken^5,7^) to 1 year after diagnosis, the date of death, or the end of the study (June 26, 2021), whichever occurred first. Hence, all patients in the cohort had a minimum of 6-month follow-up from the date of diagnosis.

### Outcomes

Our primary outcome was a time-to-event variable representing the number of days from the date of cancer diagnosis to the date of first receipt of surgery, chemotherapy, or radiation. Patients who were alive and untreated at the end of the follow-up window were censored. To determine each patient’s modality of first cancer treatment, we obtained surgical procedure records from CIHI and then matched them to OCR diagnosis data with respect to surgical site and cancer type; chemotherapy and radiation visits were defined using physician billing records from the OHIP claims database.^2,18^ We did not consider hormonal therapy for breast or prostate cancer due to insufficient capturing of this procedure at the time of analysis.^19^ For patients who were treated within 6 months after diagnosis, we analyzed their time to first cancer treatment (ie, the waiting time) as a secondary continuous outcome in a descriptive analysis. We chose 6 months (rather than 1 year) to extract the waiting time statistics because the treatment status of the entire cohort was fully observed up to this point, which reduced the potential selection bias.

### Exposure

Our primary binary exposure was whether patients received their cancer diagnosis during the prepandemic or the pandemic period. We used March 15, 2020, to represent the start date of COVID-19 in Ontario, when hospitals were directed by the Chief Medical Office of Health to discontinue nonemergent and elective procedures.^20^ As such, the prepandemic period was from January 3, 2016, to March 14, 2020, and the pandemic period was from March 15, 2020, to November 7, 2020.

### Covariates

All covariates were measured at the time of cancer diagnosis (ie, baseline). Age and sex were obtained from the RPDB. Rurality was defined as living in rural areas or small towns with an urban population of less than 10 000.^21^ Immigrant patients were identified as individuals with a record at the IRCC Permanent Resident Database. Material deprivation was reported in quintiles. Comorbidity was represented by the Elixhauser Comorbidity Index, using hospitalization records from the past 5 years.^22^ We created comorbidity groups for patients who scored 0, 1, 2, and 3 or greater on the index and for those who were not hospitalized in the past 5 years.^2,7,8,18,23^ Cancer type was determined from the OCR diagnosis records.

### Statistical Analysis

We compared the baseline characteristics of patients diagnosed in the prepandemic and the pandemic periods using 0.10 as a standardized difference threshold to identify a significant difference.^24^ For the entire cohort, we examined time to first cancer treatment in a competing risks framework. Specifically, for each treatment modality, we computed its cumulative incidence function (CIF) by considering death from any cause (30 055 deaths [9.6%] occurred during follow-up) and receipt of an alternative treatment modality as competing risks.^25^ The Gray test was performed to compare the CIF by COVID-19 period.^26^ To quantify the association between COVID-19 period and time to each modality of first cancer treatment, we constructed separate multivariable Fine-Gray subdistribution hazards models, adjusting for baseline characteristics decided a priori using both expert opinions and the literature.^27^ To quantify the differential association of the pandemic with the receipt of treatment by cancer type, we added interaction terms between the COVID-19 period exposure variable and each cancer type to these multivariable models. The results are reported as subdistribution hazard ratios (sHRs) with 95% CIs.

In a secondary analysis conducted on the subcohort that was treated within 6 months after diagnosis, we compared the mean wait times for each treatment modality by COVID-19 period using the Wilcoxon rank sum test.^28^ Analyses were 2-sided, using *P* < .05 to indicate statistical significance. We performed all analyses on SAS Enterprise Guide version 7.15 (SAS Institute).

## Results

The final cohort comprised 313 499 patients with a cancer diagnosis, of whom 29 602 (9.4%) were diagnosed in the pandemic period (Table 1). The mean (SD) age at diagnosis was 66.4 (14.1) years. A total of 153 679 patients (49.0%) were male, 39 653 (12.6%) were rural residents, and 38 866 (12.4%) were immigrant patients. We did not identify any statistically significant difference of baseline characteristics between patients diagnosed in the pandemic and prepandemic periods.

**Table 1.** Baseline Characteristics of 313 499 Patients Diagnosed With Cancer in Ontario, Canada, From January 3, 2016, to November 7, 2020^a^

Characteristic	Patients with cancer diagnosis, No. (%)		Standardized difference
	Prepandemic period (n = 283 897 [90.6%])	Pandemic period (n = 29 602 [9.4%])	
Age at diagnosis, mean (SD), y	66.38 (14.09)	66.17 (14.16)	0.01
Sex			
Female	144 755 (51.0)	15 065 (50.9)	0
Male	139 142 (49.0)	14 537 (49.1)	0
Rural^b^	35 712 (12.6)	3941 (13.3)	0.02
Immigrant	35 152 (12.4)	3714 (12.5)	0
Material deprivation, quintile^b^^,^^c^			
First, least deprived	60 230 (21.2)	6450 (21.8)	0.01
Second	58 065 (20.5)	6065 (20.5)	0
Third	54 770 (19.3)	5783 (19.5)	0.01
Fourth	54 509 (19.2)	5577 (18.8)	0.01
Fifth, most deprived	53 969 (19.0)	5465 (18.5)	0.01
Residential region			
Toronto	22 965 (8.1)	2217 (7.5)	0.02
Central	82 566 (29.1)	8631 (29.2)	0
East	73 073 (25.7)	7553 (25.5)	0.01
North	20 000 (7.1)	2185 (7.4)	0.01
West	85 271 (30.0)	9014 (30.4)	0.01
Comorbidity grouping^d^			
0	27 086 (9.5)	3091 (10.4)	0.03
1	22 647 (8.0)	2367 (8.0)	0
2	16 832 (5.9)	1533 (5.2)	0.03
≥3	24 422 (8.6)	2190 (7.4)	0.04
No hospitalization	192 910 (68.0)	20 421 (69.0)	0.02
Cancer type			
Breast	44 135 (15.5)	4546 (15.4)	0.01
Central nervous system	3796 (1.3)	467 (1.6)	0.02
Cervical	2297 (0.8)	239 (0.8)	0
Colorectal	32 249 (11.4)	3352 (11.3)	0
Endocrine	11 468 (4.0)	1075 (3.6)	0.02
Esophagus	2938 (1.0)	380 (1.3)	0.02
Genitourinary	20 866 (7.3)	2417 (8.2)	0.03
Gynecologic, excluding cervical	15 681 (5.5)	1739 (5.9)	0.02
Head and neck	8843 (3.1)	979 (3.3)	0.01
Hepatobiliary	14 438 (5.1)	1523 (5.1)	0
Lung	36 431 (12.8)	3780 (12.8)	0
Lymphoma	15 924 (5.6)	1523 (5.1)	0.02
Prostate	35 200 (12.4)	3448 (11.6)	0.02
Sarcoma	4205 (1.5)	445 (1.5)	0
Stomach	5132 (1.8)	536 (1.8)	0
Other^e^	30 294 (10.7)	3153 (10.7)	0
First cancer treatment received^f^			
Surgery	122 948 (43.3)	12 419 (42.0)	0.03
Chemotherapy	48 006 (16.9)	6010 (20.3)	0.09
Radiation	43 357 (15.3)	5016 (16.9)	0.05
Untreated	69 586 (24.5)	6157 (20.8)	0.09

^a^ March 15, 2020, was used to represent the start of the COVID-19 pandemic, as Ontario hospitals were advised to cancel nonemergent and elective procedures. The prepandemic period refers to the study period before March 15, 2020 (from January 3, 2016, to March 14, 2020) and the pandemic period is the period thereafter (from March 15 to November 7, 2020). The threshold for the standardized difference to identify a statistically significant imbalance in the distribution of baseline characteristics between the 2 groups was set at 0.10.

^b^ Overall, 0.2% and 0.8% of patients in the cohort had unknown rural living and material deprivation data, respectively. Pattern of missingness did not differ between patients in prepandemic and the pandemic periods (both standardized differences <0.02). These patients were excluded from the subsequent multivariable regression analysis.

^c^ Material deprivation was derived from the Ontario Marginalization Index and reported in quintiles. It encompasses the proportion of population that is without a high school degree, unemployed, and low-income and families that are single parent, receiving government transfer payments, and living in a dwelling in need of a major repair.

^d^ The Elixhauser Comorbidity Index is a well-validated measure of comorbidity. We computed this index using hospitalization records over the 5 years leading to the date of cancer diagnosis.

^e^ Patients with skin cancer (807), melanoma (15 480), paraneoplastic neurological syndromes (25), and ophthalmologic cancer (120) were excluded from the study cohort. Cancer types captured in other are presented in eTable 2 in Supplement 1.

^f^ Patients were followed up from date of cancer diagnosis to 1 year after diagnosis, date of death, or June 26, 2021, whichever occurred first.

**Figure 1.**
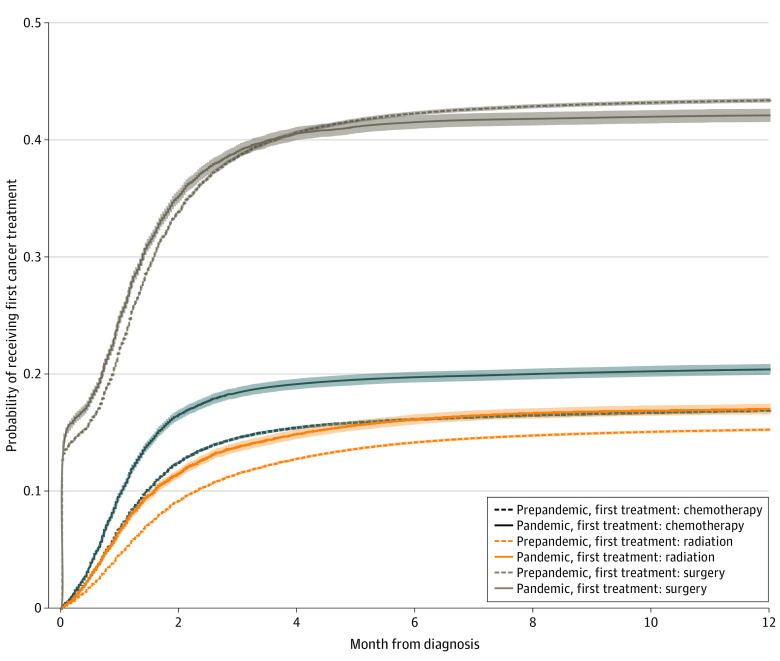
Cumulative Incidence Functions of First Cancer Treatment Received Within 1 Year After Diagnosis by Modality and COVID-19 Pandemic Period The cumulative incidence function and associated 95% CIs (represented by shaded areas) of each modality of first cancer treatment were estimated by modeling death without receiving any treatment (not plotted) and the receipt of an alternative modality of first treatment as competing risks. Using the Gray test, the cumulative incidence functions differed significantly by COVID-19 period for chemotherapy and radiation (both *P* < .001) but did not differ for surgery (*P* = .12).

During both periods, we found surgery to be the most frequently received modality of first cancer treatment, followed by chemotherapy, and lastly, radiation therapy, within the first year after diagnosis (Figure 1). The CIFs of surgery did not vary significantly between the 2 periods (Gray test *P* = .12), while the CIFs of both chemotherapy and radiation rose in the pandemic period (both Gray test *P* < .001). Six months from the date of cancer diagnosis, at which point we fully observed the treatment status of the entire cohort, we estimated the probability of first receiving surgery to decrease insignificantly from 42.2% (95% CI, 42.0%-42.4%) to 41.4% (95% CI, 40.9%-42.0%), while the probability of first receiving chemotherapy or radiation increased significantly from 16.1% (95% CI, 16.0%-16.3%) to 19.8% (95% CI, 19.3%-20.2%) and from 14.2% (95% CI, 14.1%-14.3%) to 16.2% (95% CI, 15.7%-16.6%), respectively.

In the multivariable regression analysis, we found patients diagnosed with cancer in the pandemic period were less likely to receive surgery as an initial treatment (sHR, 0.97; 95% CI, 0.95-0.99) and more likely to receive chemotherapy (sHR, 1.26; 95% CI, 1.23-1.30) or radiation therapy (sHR, 1.16; 95% CI, 1.13-1.20) as first treatment compared with their prepandemic counterparts (Table 2; eTable 3 in Supplement 1). We also found other factors to be independently associated with the receipt of first cancer treatment: immigrant patients were less likely to receive surgery or radiation as an initial treatment, but not chemotherapy; those in the lowest material deprivation quintile (indicating the highest socioeconomic status) were more likely to first receive surgery or chemotherapy and less likely to be treated by radiation first.

**Table 2.** Multivariable Competing Risks Regression Results Showing the Pandemic Impact on the Receipt of First Cancer Treatment^a^

Variable	Modality of first cancer treatment received within 1 y after cancer diagnosis					
	Surgery		Chemotherapy		Radiation therapy	
	sHR (95% CI)	*P* value	sHR (95% CI)	*P* value	sHR (95% CI)	*P* value
Cancer diagnosed in the pandemic vs prepandemic period	0.97 (0.95-0.99)	.001	1.26 (1.23-1.30)	<.001	1.16 (1.13-1.20)	<.001
Age at diagnosis, each 10-y increase	0.941 (0.938-0.945)	<.001	0.844 (0.839-0.849)	<.001	1.09 (1.08-1.10)	<.001
Female vs male patients	1.11 (1.09-1.12)	<.001	0.94 (0.93-0.96)	<.001	0.86 (0.84-0.88)	<.001
Immigrant vs nonimmigrant patients	0.96 (0.94-0.97)	<.001	1.01 (0.99-1.04)	.36	0.85 (0.82-0.87)	<.001
Material deprivation (vs fifth quintile, most deprived)^b^						
First, least deprived	1.07 (1.05-1.08)	<.001	1.086 (1.057-1.116)	<.001	0.92 (0.90-0.95)	<.001
Second	1.06 (1.05-1.08)	<.001	1.089 (1.060-1.119)	<.001	0.93 (0.91-0.96)	<.001
Third	1.05 (1.03-1.07)	<.001	1.06 (1.03-1.09)	<.001	0.95 (0.92-0.98)	<.001
Fourth	1.03 (1.01-1.05)	.001	1.03 (1.00-1.06)	.02	0.96 (0.94-0.99)	.007
Comorbidity (vs no hospitalization)^c^						
0	0.99 (0.97-1.01)	.31	1.05 (1.03-1.08)	<.001	0.91 (0.89-0.94)	<.001
1	0.94 (0.92-0.96)	<.001	0.99 (0.95-1.02)	.37	0.95 (0.92-0.98)	.002
2	0.88 (0.86-0.91)	<.001	0.86 (0.82-0.89)	<.001	0.97 (0.93-1.01)	.11
≥3	0.74 (0.72-0.75)	<.001	0.7 (0.68-0.73)	<.001	0.96 (0.92-0.99)	.007

Abbreviation: sHR, subdistribution hazard ratio.

^a^ Death from any cause and receiving either of the other 2 modalities of first treatment were modeled as competing risks. This model also adjusted for cancer type using breast cancer as the reference level (results reported in eTable 3 in Supplement 1). March 15, 2020, was used to define the prepandemic period (January 3, 2016, to March 14, 2020) and the pandemic period (March 15 to November 7, 2020).

^b^ In all 3 regression analyses, the 4 material deprivation variables were jointly significant (type III *P* < .001), indicating the presence of an overall association between material deprivation and the subdistribution hazards of receiving each modality of first cancer treatment.

^c^ In all 3 regression analyses, the 4 comorbidity variables were jointly significant (type III *P* < .001), indicating the presence of an overall association between comorbidity and the subdistribution hazards of receiving each modality of first cancer treatment.

**Figure 2.**
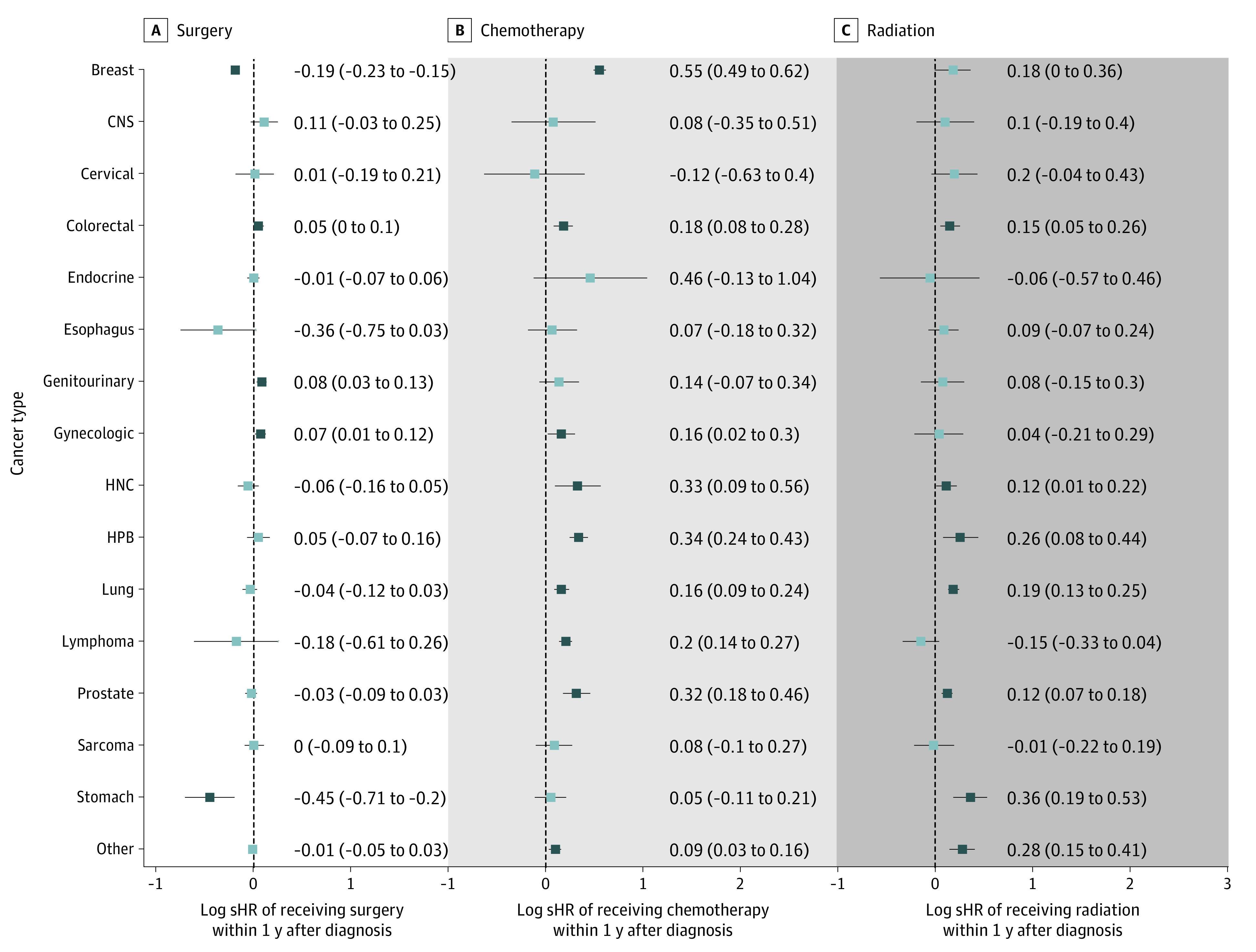
Cancer Type–Specific Subdistribution Hazard Ratios (sHRs) of Receiving Each Modality of First Cancer Treatment by COVID-19 Period For each modality of cancer treatment, sHRs and associated 95% CIs are reported on a log scale from the multivariable Fine-Gray regression model, where interaction of the pandemic indicator (pandemic vs prepandemic) with cancer type (vs breast cancer) was included. A log(sHR) greater than 0 indicates an increased rate of being first treated by this modality within 1 year after cancer diagnosis in the pandemic relative to prepandemic period. We present each sHR and associated 95% CI in eTable 4 in Supplement 1. Cancer types captured in other are presented in eTable 2 in Supplement 1. CNS indicates central nervous system; HNC, head and neck; HPB, Hepato-pancreatic-biliary.

To quantify the association between the pandemic and the receipt of treatment by cancer type, we added interaction terms between the COVID-19 indicator and cancer type to the multivariable model (Figure 2; eTable 4 in Supplement 1). We found patients with breast (sHR, 0.83; 95% CI, 0.80-0.86) and gastric (sHR, 0.64; 95% CI, 0.49-0.82) cancers to be less likely to receive surgery first during the pandemic vs prepandemic periods. Instead, patients with breast cancer were more likely to receive chemotherapy first (sHR, 1.74; 95% CI, 1.63-1.85), while patients with gastric cancer were more likely to undergo radiation therapy first (sHR, 1.43; 95% CI, 1.20-1.71).

A secondary analysis was conducted to assess the waiting times of patients who were treated within 6-month postdiagnosis (228 755 patients [73.0%]) (Table 3). Overall, we found the mean waiting times for each modality of first cancer treatment to be significantly shorter in the pandemic period than before. Specifically, the mean (SD) waiting times for surgery as an initial treatment decreased the most by 16.0% (from 35.1 [37.2] days to 29.5 [33.6] days), followed by radiation (12.2%, from 55.8 [41.8] days to 49.0 [40.1] days), and lastly, chemotherapy (12.1%, from 43.7 [34.1] days to 38.4 [30.6] days). Overall, the mean (SD) waiting times from cancer diagnosis to receiving any treatment decreased by 12.4% (from 41.0 [38.3] days to 35.9 [35.2] days) in the pandemic.

**Table 3.** Summary of Waiting Times for First Cancer Treatment Among 228 755 Patients Who Were Treated Within 6 Months After Cancer Diagnosis^a^

Modality and period	No. of recipients	Waiting times, mean (SD), d	*P* value
Surgery			
Prepandemic	119 777	35.1 (37.2)	<.001
Pandemic	12 267	29.5 (33.6)	
Chemotherapy			
Prepandemic	45 807	43.7 (34.1)	<.001
Pandemic	5846	38.4 (30.6)	
Radiation			
Prepandemic	40 277	55.8 (41.8)	<.001
Pandemic	4781	49.0 (40.1)	
Any treatment			
Prepandemic	205 861	41.0 (38.3)	<.001
Pandemic	22 894	35.9 (35.2)	

^a^ The prepandemic period is from January 3, 2016, to March 14, 2020, while the pandemic period is from March 15 to November 7, 2020. Period refers to when patients received the cancer diagnosis. For each modality of first cancer treatment, we conducted a Wilcoxon rank sum test to compare the mean duration of waiting times by COVID-19 period.

## Discussion

In this population-based study, we found the patterns of first cancer treatment, including modalities and wait times, to be associated with significant changes with the pandemic. Patients diagnosed during the pandemic were less likely to receive surgery as an initial treatment compared with their prepandemic counterparts; concomitantly, use of chemotherapy or radiation therapy as first treatment increased. When stratified by cancer type, the lower receipt of surgery as an initial treatment was significant for breast and gastric cancers. Among those who were treated 6 months after the diagnosis, the mean wait times for each treatment modality were reduced in the pandemic. These results must be interpreted in the context of a lower reported cancer incidence and disruptions in cancer screening, staging, and other activities.

Our analysis was in line with the provincial recommendation whereby physicians were directed to prioritize using nonsurgical cancer treatment at the beginning of COVID-19.^10,11^ Furthermore, for patients who did receive surgery as an initial treatment in 6 months, those diagnosed during the pandemic were operated on 5.6 days faster than their prepandemic counterparts, which could be attributed to a lower demand for cancer surgery and directed prioritization of health care resources, including imaging for cancer symptom assessment and surgical care for patients with cancer over nononcologic elective surgeries.^7,10,11,23^ The diversion of surgical resources to cancer has inevitably and negatively affected other noncancer sectors, but the exact magnitude and consequences of this decision on patients and the system are unclear. Thus, future work is required to assess the relative harms of cancelling or delaying cancer treatment, especially surgery, compared with interventions for patients without cancer. While a robust ethical framework exists for these decisions,^10,11,29^ a lack of data on the negative consequences of delaying elective benign surgery on patients makes it hard to place a relative value on one procedure over the other. While these decisions seem to have excellent face validity, extended delays in surgery of all varieties are problematic, particularly when a health care system is unable to ramp up surgical activity during the recovery phase to extend beyond typical volume.^18^

As for nonsurgical modalities, our analysis suggests patients were both more likely to get them as first cancer treatments and initiated those treatments more expeditiously in the pandemic. While this likely reflects limitations in surgical resources with stringent prioritization strategies, it may also point to an increase in the appropriate receipt of neoadjuvant therapy that the pandemic has paradoxically brought about. Patients who were offered this treatment approach sometimes rejected it to opt for direct surgery, but due to the limited surgical capacity and reluctance to undergo operations during the pandemic,^30^ these patients may have embraced this care pathway to a larger extent. Future research needs to examine these speculations by following up patients beyond the receipt of initial therapy to identify their subsequent treatment and long-term outcomes.^19,23^

Beyond the directed changes in physician practice, underlying shifts in the patient case-mix may have contributed to the different cancer treatment patterns observed in the pandemic. Particularly, the emergency cessation of cancer screening, in-person oncologist visits, and biopsies has potentially resulted in an upstage migration. However, the literature suggests the extent of such a migration, at least in the early pandemic, may be smaller than expected.^12,31,32,33^ Still, some evidence suggests an increased presentation of advanced cancers during the pandemic,^9^ many of which were inoperable and necessitated a nonsurgical approach. As such data are not yet available in our cancer registry, future research needs to assess to what extent has stage migration shifted the treatment approaches for newly diagnosed cancers in the pandemic.

We found gastric and breast cancers to be the only cancer types associated with a lower receipt of surgery as an initial treatment in the pandemic, possibly because of reasonable neoadjuvant options for these cancer types. Gastric surgery requires extensive multidisciplinary discussion and accurate staging, and small disruptions of these activities may have reduced surgery volume.^34^ We found more receipt of radiotherapy, not chemotherapy, as an initial treatment for gastric cancer in the pandemic, although a neoadjuvant radiotherapy regimen for this cancer type has not been formally established.^35,36^ This raises concerns regarding the unknown treatment side effects that would require physicians to closely monitor the patients who were treated in a nonstandard fashion. For breast cancer, the reduced receipt of surgery as an initial treatment was accompanied by a rise in use of chemotherapy as an initial treatment. This may be linked to the 3-month suspension of Ontario’s breast screening program with participation restored a year later.^6^ As such, breast cancer incidence has dropped considerably, and for those who did get a diagnosis, preliminary data suggest that many were at a more advanced stage.^37^ For patients with early-stage disease, an increasing number of them received neoadjuvant chemotherapy, as surgery was postponed.^13,19^ These findings highlight the heterogenous consequences of the pandemic on different types of cancer. Modeling studies aimed at forecasting long-term cancer-related outcomes, such as excess cancer deaths, by cancer type could use our data to improve their estimates.^37,38^

We also found immigrant status and material deprivation to be independently and negatively associated with the receipt of surgery as an initial cancer treatment in both prepandemic and pandemic periods. Immigrant patients may lack a strong support system, such as affiliation with a family doctor, causing oncologists to postpone the discussion about treatment.^39^ In terms of material deprivation, it is possible that patients belonging to a higher socioeconomic class were able to advocate for having the standard curative treatment (surgery) to a larger extent than those with a lower status. Future research needs to examine whether these inequalities in cancer care were accentuated during the pandemic.^23^

### Limitations

This study has limitations. Our results are subject to potential unmeasured confounding bias particularly related to cancer staging and subsites, as these data will take time to accrue in our cancer registry. Future studies should replicate our analysis for each cancer type, where a more granular analysis may be required for breast and gastric cancers. Next, we were unable to track whether further shifts in first cancer treatment occurred beyond when our cancer registry was last updated. Particularly, the second, third, and fourth waves of COVID-19 peaked in Ontario in January 2021, April 2021, and January 2022, respectively^40^; these pandemic-related milestones need to be explicitly incorporated into future analyses.^18^ Additionally, 27% of our cohort (84 744 patients) was untreated in the first 6 months after diagnosis. There may be more nuanced shifts within this large group that are worthy of further study. For example, with staging data, future studies can identify patients initiating palliative care on diagnosis. The untreated cohort in this study also included patients receiving hormonal therapy, a procedure that we were unable to explicitly and comprehensively capture.^19^ Future research will allow us to provide data on the initiation of hormonal therapy in the pandemic compared with prepandemic times.

## Conclusions

In this population-based cohort study involving all new cancer diagnoses from 2016 to 2020 in Ontario, Canada, we found that being diagnosed in the COVID-19 pandemic was associated with lower receipt of surgery as an initial cancer treatment during the first year of diagnosis. Patients with breast cancer experienced an increased receipt of chemotherapy as first treatment during the pandemic. Among those who were treated within 6 months after diagnosis, their mean waiting times decreased by 12% to 16% in the pandemic, depending on the modality of treatment received. These findings highlight the importance of maintaining timely access of treatment for patients with new cancer diagnoses in an overwhelmed health care system. Future work needs to examine how these changes may have affected patient outcomes to inform future pandemic guideline development.

## Notes

Supplement 1. **eFigure. **Flow Diagram Showing the Creation of the Study Cohort **eTable 1. **Data Sources **eTable 2. **Identifying Cancer Types From the Ontario Cancer Registry (OCR) **eTable 3. **Complete Results of the Multivariable Competing Risk Model Examining Receipt of First Cancer Treatment **eTable 4. **Cancer Type–Specific Hazard Ratios Computed From the Multivariable Model With Interaction Effects

Supplement 2.
Data Sharing Statement
