# Supplementary material for: Timeliness and Modality of Treatment for New Cancer Diagnoses During the COVID-19 Pandemic in Canada
Source: JAMA Netw Open. 2023 Jan 10;6(1):e2250394. doi: 10.1001/jamanetworkopen.2022.50394 (PMC9856765; doi:10.1001/jamanetworkopen.2022.50394)
Supplement: Supplement 1. — eFigure. Flow Diagram Showing the Creation of the Study Cohort eTable 1. Data Sources eTable 2. Identifying Cancer Types From the Ontario Cancer Registry (OCR) eTable 3. Complete Results of the Multivariable Competing Risk Model Examining Receipt of First Cancer Treatment eTable 4. Cancer Type–Specific Hazard Ratios Computed From the Multivariable Model With Interaction Effects [file jamanetwopen-e2250394-s001.pdf]

Supplemental Online Content

Fu R, Sutradhar R, Li Q, et al. Timeliness and modality of treatment for new cancer diagnoses during the COVID-19 pandemic in Canada. *JAMA Netw Open.* 2023;6(1):e2250394. doi:10.1001/jamanetworkopen.2022.50394

eFigure. Flow Diagram Showing the Creation of the Study Cohort

eTable 1. Data Sources

eTable 2. Identifying Cancer Types From the Ontario Cancer Registry (OCR)

eTable 3. Complete Results of the Multivariable Competing Risk Model Examining Receipt of First Cancer Treatment

eTable 4. Cancer Type–Specific Hazard Ratios Computed From the Multivariable Model With Interaction Effects

This supplemental material has been provided by the authors to give readers additional information about their work.

© 2023 Fu R et al. *JAMA Network Open.*

eFigure. Flow Diagram Showing the Creation of the Study Cohort

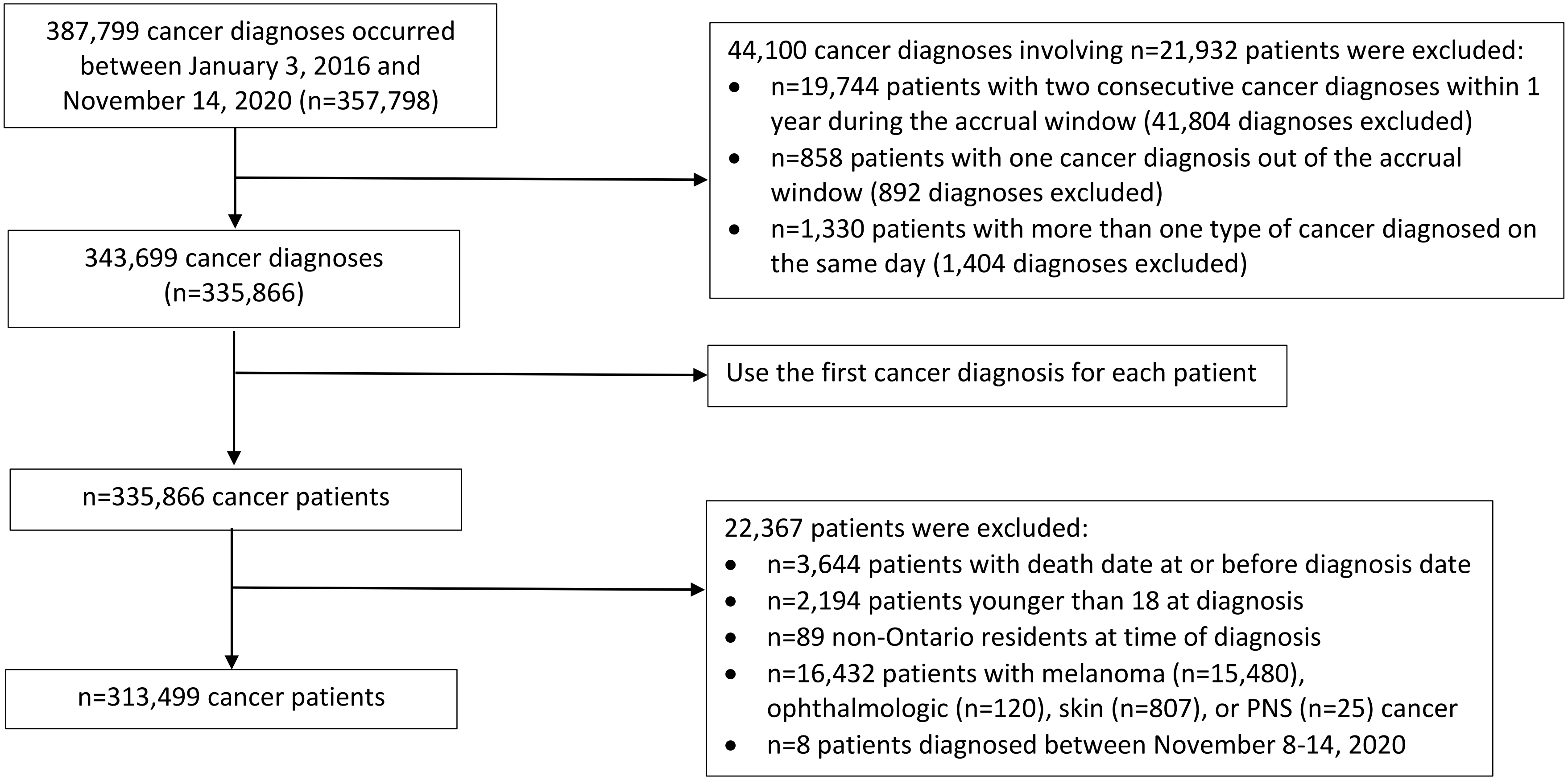

The number of patients (n) at each stage of exclusion is reported in the parentheses. We excluded patients with a diagnosis of melanoma or skin cancer to ensure a robust capture of cancer surgeries performed at hospital since these cancers are frequently treated in a clinic setting. We also excluded ophthalmologic and paraneoplastic neurological syndromes (PNS) cancer patients due to their small numbers. Because the Ontario Cancer Registry (OCR) database had reliable data until November 7, 2020 at the time of this analysis (January 2022), we excluded the 8 patients who were diagnosed in November 8-14, 2020.

***Abbreviation**:* PNS, paraneoplastic neurological syndromes.

© 2023 Fu R et al. *JAMA Network Open.*

eTable 1. Data Sources

Database	Description
**Ontario Cancer Registry (OCR)**	The OCR is the provincial registry of all incident cancer diagnoses in Ontario with a capture rate of 96%. Information included in the registry: cancer topography and morphology/histology, and details on diagnosis (e.g., types of contributing information to the diagnosis, dates), and patients who have died of cancer.
**Ontario Health Insurance Plan (OHIP) Claims Database**	The OHIP claims database contains all physician billing records including information on diagnoses and services provided, such as receipt of chemotherapy and radiotherapy.<br>Chemotherapy: G339, G345, G359, G381, G281, G382, G388<br>Radiotherapy: X310, X311, X312, X313, X302, X304, X305, X306, X322, X323, X334, X324, X325, X326, X327, X335, X328, X329, X332, X336, X330
**Discharge Abstract Database (DAD) and Same Day Surgery (SDS)**	The DAD and SDS are both data holdings of the Canadian Institute for Health Information (CIHI) that capture administrative, clinical, and demographic information on hospital discharges (including deaths, sign-outs, and transfers) and day surgery.
**Registered Persons Database (RPDB)**	The RPDB is an ICES database derived from all administrative data sources and provides demographic data including age, patient residence, vital status, date of last contact with the healthcare system, and OHIP eligibility.
**Immigration, Refugees and Citizenship Canada (IRCC) Permanent Residents database**	The IRCC Permanent Resident Database (with data from January 1985 to May 2017) includes records of individuals who immigrated to Ontario during this period.
**Ontario Marginalization Index (ONMARG)**	The ONMARG is a specialized database using census data to profile relative area-level marginalization dependency, deprivation, ethnic concentration, and instability at various geographic levels in Ontario. The material deprivation dimension captures the proportion of population that is without a high school degree, unemployed, low-income, single-parent families, receiving government transfer payments, and living in dwelling in need of a major repair.
**Ontario Registrar General (ORG)**	The ORG contains gold standard vital status data for all Ontarians. According to the Vital Statistics Act, it is mandatory to register all deaths occurring in the province.

© 2023 Fu R et al. *JAMA Network Open.*

eTable 2. Identifying Cancer Types From the Ontario Cancer Registry (OCR)

Cancer site	ICD-O-3 code
Breast	C50
Central nervous system	C70.0, C70.1, C70.9, C71, C72
Colorectal	C17, C18, C19.9, C20.9, C21.0, C21.1, C21.2, C21.8
Cervical	C53.0, C53.1, C53.8, C53.9
Endocrine	C73.9, C74.0, C74.1, C74.9, C75
Esophagus	C15
Genitourinary	C60, C62, C64, C65, C66, C67, C68
Gynecological exclude cervical	C51, C52, C54, C55, C56, C57
Head and neck	C10.0, C10.1, C10.2, C10.3, C10.4, C10.8, C10.9, C01.9, C02.0, C02.1, C02.2, C02.3, C02.4, C02.8, C02.9, C03.0, C03.1, C03.9, C04.0, C04.1, C04.8, C04.9, C05.0, C05.1, C05.2, C05.8, C05.9, C06.0, C06.1, C06.2, C06.8, C06.9, C07.9, C08.0, C08.1, C08.8, C08.9, C09.0, C09.1, C09.8, C09.9, C11.0, C11.1, C11.2, C11.3, C11.8, C11.9, C12.9 , C14.0, C14.2, C14.8, C76.0, C06.9, C14.8, C32.0, C32.1, C32.3, C32.8, C32.9, C13.0, C13.1, C13.2, C13.8, C13.9, C00.0, C00.1, C00.2, C00.3, C00.4, C00.5, C00.6, C00.8, C00.9, C14.8, C44.0
Hepatic, pancreatic or biliary	C22.0, C22.1, C23, C24, C25
Lung	C34
Lymphoma	C77
Prostate	C61.9
Sarcoma	C00.0, C00.1, C00.3, C00.5, C00.9, C01.9 to C02.3, C02.8 to C03.1, C03.9, C04.0, C04.9, C.05.0, C05.1, C05.9, C06.0, C06.2, C06.9, C07.9, C08.0, C08.9, C09.0, C09.9, C10.3, C10.9, C11.0 to C11.3, C11.8, C11.9, C13.0, C13.1, C13.8, C13.9, C14.0, C14.8, C15.0, C15.3, C15.4, C15.5, C15.9, C16.0 to C16.6, C16.8 to C17.3, C17.8 to C18.9, C19.9, C20.9, C22.0, C22.1, C23.9 to C24.1, C24.9 to C25.2, C25.9, C30.0, C30.1, C31.1 to C31.3, C31.8 to C32.3, C32.9, C33.9 to C34.3, C34.8, C34.9, C37.9 to C38.3, C40.1 to C40.3, C40.8 to C41.4, C41.9, C42.1 to C42.4, C44.0 to C44.9, C47.0 to C47.9, C49.0 to C49.9, C50.0 to C512, C51.8, C51.9, C52.9 to C53.1, C53.8 to C54.3, C54.8, C54.9, C56.9 to C57.4, C57.7 to C57.9, C60.0 to C60.2, C60.9, C61.9 to C62.1, C62.9 to C63.2, C63.7 to C63.9, C649., C65.9, C66.9 to C68.0, C68.8, C69.0, C69.3, C69.6, C69.8, C70.0, C70.1, C70.9 to C72.0, C72.5, C72.9, C73.9 to C74.1, C74.9, C75.5, C77.0 to C77.9 with morphology code 803*, 831*, 871*, 880*-885*, 890*-900*, 912*, 914*, 917*-919*, 922*-924*, 926*, 933*, 944*, 948*, 953*, 958*, 974*-975*, 993*
Stomach	C16
Other	C26.0, C26.8, C26.9, C30, C31, C32.2, C33.9, C37.9, C38, C39, C40, C41, C42.0-C42.4, C44.1, C48, C49, C58.9, C63, C76, C80.9

We did not consider melanoma and skin cancers to ensure a reliable capture of hospital-based cancer-directed surgical procedures as these cancers are frequently treated in the outpatient setting. We also did not include ophthalmologic and paraneoplastic neurological syndromes cancers as these cancers were extremely rare in our cohort (<0.04% of the cohort).

© 2023 Fu R et al. *JAMA Network Open.*

eTable 3. Complete Results of the Multivariable Competing Risk Model Examining Receipt of First Cancer Treatment

	Modality of first cancer treatment received within 1-year after cancer diagnosis								
	Surgery			Chemotherapy			Radiation therapy		
**Variables**	**sHR**	**95% CI**	**p-value**	**sHR**	**95% CI**	**p-value**	**sHR**	**95% CI**	**p-value**
**Pandemic vs. pre-pandemic**[1]	0.97	0.95-0.99	<0.01	1.26	1.23-1.30	<0.01	1.16	1.13-1.20	<0.01
**Age at diagnosis, each 10-year increase**	0.941	0.938-0.945	<0.01	0.844	0.839-0.849	<0.01	1.09	1.08-1.10	<0.01
**Females vs. males**	1.11	1.09-1.12	<0.01	0.94	0.93-0.96	<0.01	0.86	0.84-0.88	<0.01
**Immigrants vs. non-immigrants**	0.96	0.94-0.97	<0.01	1.01	0.99-1.04	0.36	0.85	0.82-0.87	<0.01
**Material deprivation (vs. 5, most)**[2]									
1, least deprived	1.07	1.05-1.08	<0.01	1.086	1.057-1.116	<0.01	0.92	0.90-0.95	<0.01
2	1.06	1.05-1.08	<0.01	1.089	1.060-1.119	<0.01	0.93	0.91-0.96	<0.01
3	1.05	1.03-1.07	<0.01	1.06	1.03-1.09	<0.01	0.95	0.92-0.98	<0.01
4	1.03	1.01-1.05	<0.01	1.03	1.00-1.06	0.02	0.96	0.94-0.99	<0.01
**Comorbidity (vs. no hospitalization)**[3]									
0	0.99	0.97-1.01	0.31	1.05	1.03-1.08	<0.01	0.91	0.89-0.94	<0.01
1	0.94	0.92-0.96	<0.01	0.99	0.95-1.02	0.37	0.95	0.92-0.98	<0.01
2	0.88	0.86-0.91	<0.01	0.86	0.82-0.89	<0.01	0.97	0.93-1.01	0.11
3+	0.74	0.72-0.75	<0.01	0.70	0.68-0.73	<0.01	0.96	0.92-0.99	<0.01
**Cancer type (vs. breast cancer)**[4]									
Central nervous system	1.29	1.23-1.35	<0.01	0.25	0.22-0.29	<0.01	4.32	3.86-4.83	<0.01
Cervical	0.34	0.32-0.36	<0.01	0.34	0.29-0.39	<0.01	17.61	16.06-19.31	<0.01
Colorectal	0.79	0.77-0.80	<0.01	0.74	0.71-0.77	<0.01	4.33	4.05-4.63	<0.01
Endocrine	1.76	1.73-1.80	<0.01	0.036	0.029-0.043	<0.01	0.69	0.60-0.81	<0.01
Esophagus	0.094	0.085-0.105	<0.01	1.20	1.11-1.31	<0.01	26.42	24.43-28.57	<0.01
Genitourinary	1.46	1.43-1.49	<0.01	0.22	0.21-0.24	<0.01	1.22	1.11-1.34	<0.01
Gynecologic excluding cervical	1.06	1.04-1.08	<0.01	0.69	0.66-0.73	<0.01	1.56	1.42-1.71	<0.01
Head and neck	0.42	0.41-0.43	<0.01	0.36	0.33-0.40	<0.01	18.70	17.48-20.01	<0.01
Hepato pancreatic biliary	0.205	0.197-0.213	<0.01	1.85	1.78-1.92	<0.01	2.64	2.43-2.86	<0.01
Lung	0.204	0.199-0.209	<0.01	1.26	1.22-1.30	<0.01	17.88	16.81-19.01	<0.01
Lymphoma	0.014	0.013-0.016	<0.01	4.26	4.12-4.39	<0.01	3.38	3.13-3.65	<0.01
Prostate	0.317	0.309-0.324	<0.01	0.29	0.27-0.30	<0.01	10.69	10.03-11.40	<0.01
Sarcoma	0.00	0-0	<0.01	1.35	1.27-1.44	<0.01	9.94	9.14-10.80	<0.01
Stomach	0.17	0.16-0.18	<0.01	2.20	2.09-2.32	<0.01	9.22	8.49-10.01	<0.01
Other[4]	0.00	0-0	<0.01	2.66	2.58-2.75	<0.01	2.46	2.29-2.64	<0.01

[1] We used March 15, 2020 to define the pre-pandemic period (January 3, 2016 to March 14, 2020) and the pandemic period (March 15, 2020 to November 7, 2020). Death from any cause and receiving either of the other two modalities of first treatment were modelled as competing risks.
[2] In all three regression analyses, the four material deprivation variables were jointly significant (Type-III p-value < 0.01), indicating the presence of an overall association between material deprivation and the subdistribution hazards of receiving each modality of first cancer treatment.
[3] In all three regression analyses, the four comorbidity variables were jointly significant (Type-III p-value < 0.01), indicating the presence of an overall association between comorbidity and the subdistribution hazards of receiving each modality of first cancer treatment.
[4] Cancer types captured in “Other” are detailed in **eTable 2**. ***Abbreviations***: sHR, subdistribution hazard ratio; CI, confidence interval.

© 2023 Fu R et al. *JAMA Network Open.*

eTable 4. Cancer Type–Specific Hazard Ratios Computed From the Multivariable Model With Interaction Effects

	Modality of first cancer treatment received within 1-year after cancer diagnosis								
**Pandemic vs. pre-pandemic**	**Surgery**			**Chemotherapy**			**Radiation therapy**		
**Cancer type**	**sHR**	**95% CI**	**p-value**	**sHR**	**95% CI**	**p-value**	**sHR**	**95% CI**	**p-value**
Breast	0.83	0.80-0.86	<0.01	1.74	1.63-1.85	<0.01	1.20	1.00-1.44	0.06
Central nervous system	1.11	0.97-1.28	0.13	1.08	0.70-1.67	0.72	1.11	0.83-1.49	0.49
Cervical	1.01	0.83-1.23	0.93	0.89	0.53-1.49	0.66	1.22	0.96-1.54	0.10
Colorectal	1.05	1.00-1.11	0.04	1.20	1.08-1.32	<0.01	1.17	1.05-1.29	<0.01
Endocrine	0.99	0.93-1.06	0.87	1.58	0.88-2.83	0.12	0.95	0.57-1.58	0.83
Esophagus	0.70	0.47-1.03	0.07	1.07	0.83-1.38	0.59	1.09	0.93-1.27	0.28
Genitourinary	1.08	1.03-1.14	<0.01	1.15	0.94-1.41	0.19	1.08	0.86-1.35	0.50
Gynecologic excluding cervical	1.07	1.01-1.13	0.02	1.17	1.02-1.35	0.02	1.04	0.81-1.33	0.77
Head and neck	0.94	0.85-1.05	0.30	1.39	1.10-1.76	<0.01	1.12	1.01-1.25	0.03
Hepato pancreatic biliary	1.05	0.93-1.18	0.42	1.40	1.28-1.54	<0.01	1.30	1.08-1.56	<0.01
Lung	0.96	0.89-1.03	0.28	1.18	1.09-1.27	<0.01	1.21	1.14-1.28	<0.01
Lymphoma	0.84	0.54-1.29	0.42	1.22	1.15-1.31	<0.01	0.86	0.72-1.04	0.13
Prostate	0.97	0.92-1.03	0.36	1.37	1.19-1.58	<0.01	1.13	1.07-1.19	<0.01
Sarcoma	1.00	0.91-1.11	0.92	1.09	0.90-1.31	0.38	0.99	0.81-1.21	0.92
Stomach	0.64	0.49-0.82	<0.01	1.05	0.89-1.23	0.56	1.43	1.20-1.71	<0.01
Other	0.99	0.95-1.03	0.57	1.10	1.03-1.17	<0.01	1.32	1.16-1.50	<0.01

We report the subdistribution hazard ratios (sHRs) and associated 95% confidence intervals of each modality of first cancer treatment from the corresponding multivariable Fine-Gray regression model, where Interaction of the pandemic indicator (pandemic vs. pre-pandemic) with cancer type was included. All models also comprised the following patient-level covariates: age at cancer diagnosis, sex, immigration status, material deprivation, and comorbidity measured by the Elixhauser Comorbidity Index. Cancer types captured in “Other” are detailed in **eTable 2**. A sHR > 1 indicates an increased rate of being first treated by this modality within 1 year after cancer diagnosis in the pandemic relative to pre-pandemic era.

***Abbreviations**:* sHR, subdistribution hazard ratio; CI, confidence interval.

© 2023 Fu R et al. *JAMA Network Open.*
