# Supplementary material for: Timeliness and Modality of Treatment for New Cancer Diagnoses During the COVID-19 Pandemic in Canada
Source: JAMA Netw Open. 2023 Jan 10;6(1):e2250394. doi: 10.1001/jamanetworkopen.2022.50394 (PMC9856765; doi:10.1001/jamanetworkopen.2022.50394)
Supplement: Supplement 2. — Data Sharing Statement [file jamanetwopen-e2250394-s002.pdf]

Data Sharing Statement

Fu. Timeliness and Modality of Treatment for New Cancer Diagnoses During the COVID-19 Pandemic in Canada. *JAMA Netw Open*. Published January 10, 2023.
doi:10.1001/jamanetworkopen.2022.50394

Data
Data available: No

Additional Information
Explanation for why data not available: The dataset from this study is held securely in coded form at ICES. While legal data sharing agreements between ICES and data providers (e.g., healthcare organizations and government) prohibit ICES from making the dataset publicly available, access may be granted to those who meet pre-specified criteria for confidential access, available at www.ices.on.ca/DAS (email: das@ices.on.ca). The full dataset creation plan and underlying analytic code are available from the authors upon request, understanding that the computer programs may rely upon coding templates or macros that are unique to ICES and are therefore either inaccessible or may require modification.
